# Tetra-μ-acetato-κ^8^ *O*:*O*′-bis­{[2-(*m*-tolyl­amino)pyridine-κ*N*]copper(II)}

**DOI:** 10.1107/S1600536809050041

**Published:** 2009-11-28

**Authors:** Zainal Abidin Fairuz, Zaharah Aiyub, Zanariah Abdullah, Seik Weng Ng

**Affiliations:** a Department of Chemistry, University of Malaya, 50603 Kuala Lumpur, Malaysia

## Abstract

In the crystal structure of the title compound, [Cu_2_(C_2_H_3_O_2_)_4_(C_12_H_12_N_2_)_2_], the binuclear mol­ecule lies about a center of inversion; the four acetate groups each bridge a pair of Cu^II^ atoms. The coordination of the metal atom is distorted square-pyramidal, with the bonding O atoms comprising a square basal plane and the coordinating N atom of the *N*-heterocycle occupying the apical position. The pyridine ring is twisted with respect to the benzene ring at a dihedral angle of 45.68 (16)°. Intra­molecular N—H⋯O hydrogen bonding is present between the imino and carb­oxy groups.

## Related literature

There are many examples of tetra­kisacetatobis[(substituted pyridine)copper] complexes. For examples of 2-amino­pyridyl derivatives, see: Barquín *et al.* (2004 ▶); Seco *et al.* (2004 ▶); Sieroń (2004 ▶).
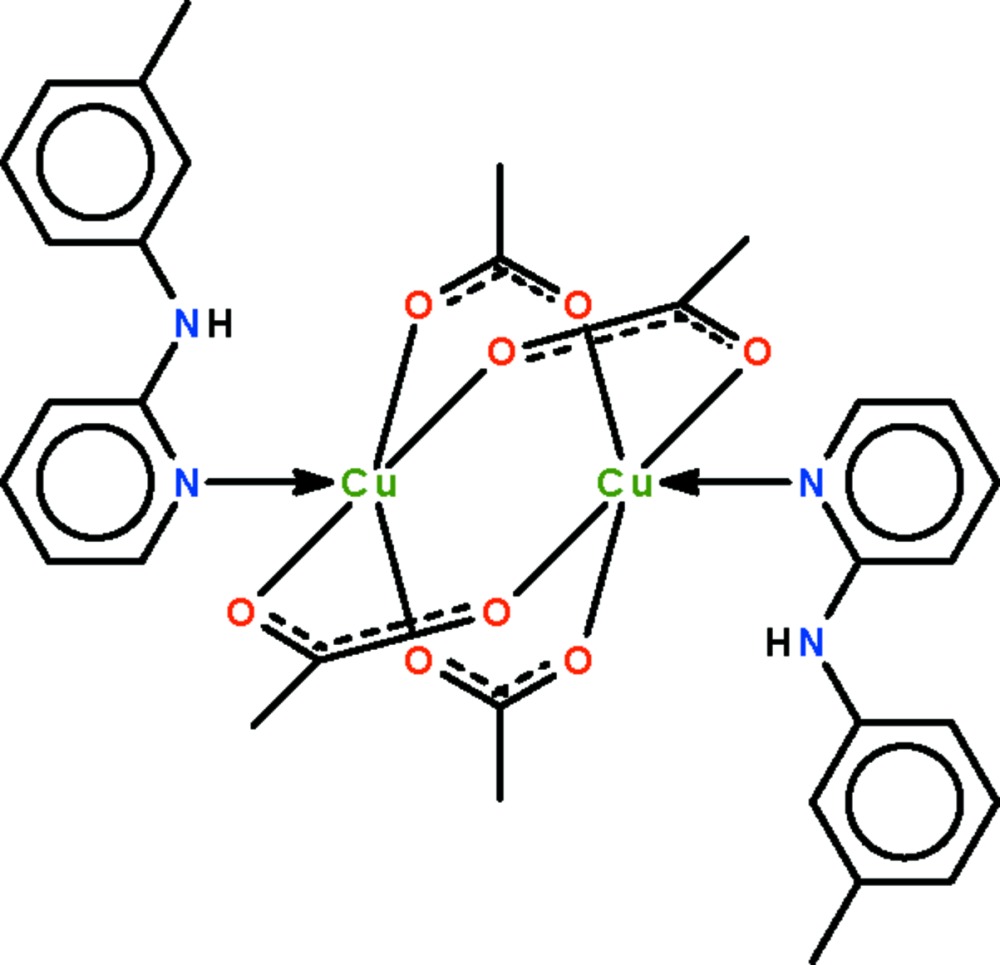


## Experimental

### 

#### Crystal data

[Cu_2_(C_2_H_3_O_2_)_4_(C_12_H_12_N_2_)_2_]
*M* *_r_* = 731.73
Triclinic, 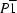

*a* = 7.7143 (2) Å
*b* = 10.5625 (3) Å
*c* = 11.2413 (3) Å
α = 66.531 (2)°
β = 85.740 (2)°
γ = 78.568 (2)°
*V* = 823.51 (4) Å^3^
*Z* = 1
Mo *K*α radiation
μ = 1.35 mm^−1^
*T* = 293 K
0.25 × 0.15 × 0.05 mm

#### Data collection

Bruker SMART APEX diffractometer
Absorption correction: multi-scan (*SADABS*; Sheldrick, 1996 ▶) *T*_min_ = 0.730, *T*_max_ = 0.936
6451 measured reflections
3678 independent reflections
2915 reflections with *I* > 2σ(*I*)
*R*_int_ = 0.023

#### Refinement

*R*[*F* ^2^ > 2σ(*F* ^2^)] = 0.038
*wR*(*F* ^2^) = 0.098
*S* = 1.07
3678 reflections
211 parameters
H-atom parameters constrained
Δρ_max_ = 0.49 e Å^−3^
Δρ_min_ = −0.59 e Å^−3^

### 

Data collection: *APEX2* (Bruker, 2008 ▶); cell refinement: *SAINT* (Bruker, 2008 ▶); data reduction: *SAINT*; program(s) used to solve structure: *SHELXS97* (Sheldrick, 2008 ▶); program(s) used to refine structure: *SHELXL97* (Sheldrick, 2008 ▶); molecular graphics: *X-SEED* (Barbour, 2001 ▶); software used to prepare material for publication: *publCIF* (Westrip, 2009 ▶).

## Supplementary Material

Crystal structure: contains datablocks global, I. DOI: 10.1107/S1600536809050041/xu2688sup1.cif

Structure factors: contains datablocks I. DOI: 10.1107/S1600536809050041/xu2688Isup2.hkl

Additional supplementary materials:  crystallographic information; 3D view; checkCIF report

## Figures and Tables

**Table 1:** Selected bond lengths (Å)

Cu1—O1	1.9762 (19)
Cu1—O2^i^	1.9866 (19)
Cu1—O3	1.967 (2)
Cu1—O4^i^	1.966 (2)
Cu1—N1	2.197 (2)

Symmetry code: (i) 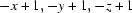.

**Table 2:** Hydrogen-bond geometry (Å, °)

*D*—H⋯*A*	*D*—H	H⋯*A*	*D*⋯*A*	*D*—H⋯*A*
N2—H2⋯O2^i^	0.86	2.17	2.913 (3)	145

Symmetry code: (i) 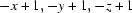.

**Fig. 1.**
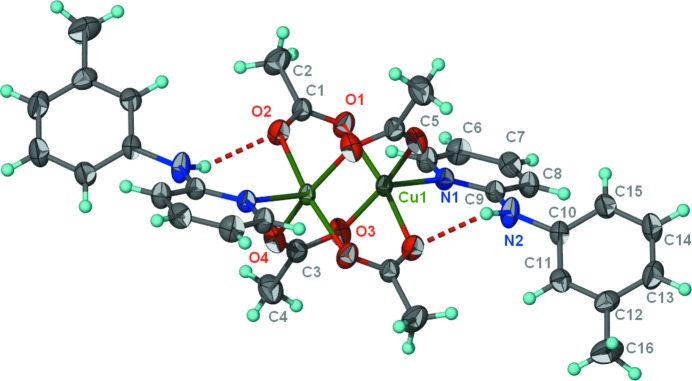
Thermal ellipsoid plot (Barbour, 2001) of Cu2(C2H3O2)4(C12H12N2)2 at the 50% probability level; hydrogen atoms are drawn as spheres of arbitrary radius. Dashed lines indicate the hydrogen bonding.

## supplementary crystallographic information

### Experimental

Copper acetate (0.1 g, 0.5 mmol) was dissolved in acetonitrile (5 ml). The solution was mixed with a solution of 3-tolylamino-2-pyridine (0.2 g, 1.1 mmol) dissolved in acetonitrile (15 ml). The green precipitate that formed was recrystallized from acetonitrile.

### Refinement

Carbon-bound H-atoms were placed in calculated positions (C–H 0.93–0.96 Å) and were included in the refinement in the riding model approximation, with *U*(H) set to 1.2–1.5*U*(C). The amino H-atom was similarly treated.

### Crystal data

**Table:**

[Cu_2_(C_2_H_3_O_2_)_4_(C_12_H_12_N_2_)_2_]	*Z* = 1
*M**_r_* = 731.73	*F*(000) = 378
Triclinic, *P*1	*D*_x_ = 1.475 Mg m^−^^3^
Hall symbol: -P 1	Mo *K*α radiation, λ = 0.71073 Å
*a* = 7.7143 (2) Å	Cell parameters from 2561 reflections
*b* = 10.5625 (3) Å	θ = 2.3–27.6°
*c* = 11.2413 (3) Å	µ = 1.35 mm^−^^1^
α = 66.531 (2)°	*T* = 293 K
β = 85.740 (2)°	Prism, green
γ = 78.568 (2)°	0.25 × 0.15 × 0.05 mm
*V* = 823.51 (4) Å^3^	

### Data collection

**Table:**

Bruker SMART APEX diffractometer	3678 independent reflections
Radiation source: fine-focus sealed tube	2915 reflections with *I* > 2σ(*I*)
graphite	*R*_int_ = 0.023
ω scans	θ_max_ = 27.5°, θ_min_ = 2.0°
Absorption correction: multi-scan (*SADABS*; Sheldrick, 1996)	*h* = −10→9
*T*_min_ = 0.730, *T*_max_ = 0.936	*k* = −13→13
6451 measured reflections	*l* = −14→14

### Refinement

**Table:**

Refinement on *F*^2^	Primary atom site location: structure-invariant direct methods
Least-squares matrix: full	Secondary atom site location: difference Fourier map
*R*[*F*^2^ > 2σ(*F*^2^)] = 0.038	Hydrogen site location: inferred from neighbouring sites
*wR*(*F*^2^) = 0.098	H-atom parameters constrained
*S* = 1.07	*w* = 1/[σ^2^(*F*_o_^2^) + (0.0426*P*)^2^ + 0.4239*P*] where *P* = (*F*_o_^2^ + 2*F*_c_^2^)/3
3678 reflections	(Δ/σ)_max_ = 0.001
211 parameters	Δρ_max_ = 0.49 e Å^−^^3^
0 restraints	Δρ_min_ = −0.59 e Å^−^^3^

### Fractional atomic coordinates and isotropic or equivalent isotropic displacement parameters (Å^2^)

**Table:**

	*x*	*y*	*z*	*U*_iso_*/*U*_eq_	
Cu1	0.58894 (4)	0.46743 (4)	0.60797 (3)	0.03044 (12)	
O1	0.6955 (3)	0.6324 (2)	0.50147 (18)	0.0439 (5)	
O2	0.5505 (3)	0.6857 (2)	0.31997 (18)	0.0419 (5)	
O3	0.7582 (3)	0.3494 (2)	0.53769 (19)	0.0460 (5)	
O4	0.6100 (3)	0.4052 (2)	0.35536 (19)	0.0436 (5)	
N1	0.7506 (3)	0.4120 (2)	0.7806 (2)	0.0304 (5)	
N2	0.5409 (3)	0.3175 (3)	0.9254 (2)	0.0446 (6)	
H2	0.4771	0.3436	0.8573	0.053*	
C1	0.6579 (4)	0.7076 (3)	0.3856 (3)	0.0342 (6)	
C2	0.7467 (5)	0.8317 (4)	0.3197 (3)	0.0570 (9)	
H2A	0.8448	0.8237	0.3717	0.085*	
H2B	0.6637	0.9162	0.3091	0.085*	
H2C	0.7885	0.8346	0.2363	0.085*	
C3	0.7354 (4)	0.3375 (3)	0.4329 (3)	0.0378 (6)	
C4	0.8709 (5)	0.2335 (4)	0.4000 (3)	0.0578 (9)	
H4A	0.9175	0.1573	0.4784	0.087*	
H4B	0.9652	0.2787	0.3520	0.087*	
H4C	0.8169	0.1979	0.3485	0.087*	
C5	0.9143 (4)	0.4436 (3)	0.7539 (3)	0.0389 (7)	
H5	0.9471	0.4835	0.6675	0.047*	
C6	1.0348 (4)	0.4198 (3)	0.8479 (3)	0.0438 (7)	
H6	1.1454	0.4446	0.8259	0.053*	
C7	0.9871 (4)	0.3582 (3)	0.9752 (3)	0.0403 (7)	
H7	1.0662	0.3400	1.0411	0.048*	
C8	0.8228 (4)	0.3236 (3)	1.0051 (3)	0.0395 (7)	
H8	0.7896	0.2817	1.0912	0.047*	
C9	0.7059 (3)	0.3518 (3)	0.9053 (2)	0.0309 (6)	
C10	0.4629 (4)	0.2444 (3)	1.0447 (2)	0.0339 (6)	
C11	0.3760 (4)	0.1388 (3)	1.0512 (3)	0.0365 (6)	
H11	0.3785	0.1149	0.9799	0.044*	
C12	0.2858 (4)	0.0682 (3)	1.1613 (3)	0.0417 (7)	
C13	0.2886 (5)	0.1024 (4)	1.2678 (3)	0.0513 (8)	
H13	0.2327	0.0539	1.3440	0.062*	
C14	0.3736 (5)	0.2078 (4)	1.2618 (3)	0.0522 (8)	
H14	0.3734	0.2302	1.3339	0.063*	
C15	0.4592 (4)	0.2808 (3)	1.1506 (3)	0.0420 (7)	
H15	0.5137	0.3533	1.1468	0.050*	
C16	0.1823 (5)	−0.0390 (4)	1.1628 (4)	0.0658 (10)	
H16A	0.2210	−0.0685	1.0932	0.099*	
H16B	0.0587	0.0016	1.1522	0.099*	
H16C	0.2012	−0.1187	1.2440	0.099*	

### Atomic displacement parameters (Å^2^)

**Table:**

	*U*^11^	*U*^22^	*U*^33^	*U*^12^	*U*^13^	*U*^23^
Cu1	0.02917 (19)	0.0399 (2)	0.02439 (17)	−0.01136 (14)	0.00032 (12)	−0.01252 (14)
O1	0.0492 (13)	0.0523 (13)	0.0312 (10)	−0.0263 (10)	−0.0027 (9)	−0.0090 (9)
O2	0.0452 (12)	0.0516 (13)	0.0325 (10)	−0.0246 (10)	−0.0018 (9)	−0.0125 (9)
O3	0.0414 (12)	0.0601 (14)	0.0384 (11)	−0.0004 (10)	−0.0021 (9)	−0.0250 (10)
O4	0.0412 (12)	0.0558 (13)	0.0376 (11)	−0.0023 (10)	−0.0020 (9)	−0.0251 (10)
N1	0.0282 (12)	0.0365 (13)	0.0277 (11)	−0.0101 (10)	−0.0003 (9)	−0.0118 (10)
N2	0.0374 (14)	0.0714 (18)	0.0249 (11)	−0.0241 (13)	−0.0002 (10)	−0.0123 (12)
C1	0.0330 (15)	0.0411 (16)	0.0309 (14)	−0.0141 (12)	0.0046 (11)	−0.0138 (12)
C2	0.065 (2)	0.058 (2)	0.0484 (19)	−0.0362 (19)	−0.0014 (16)	−0.0094 (16)
C3	0.0370 (16)	0.0418 (17)	0.0353 (15)	−0.0110 (13)	0.0082 (12)	−0.0154 (13)
C4	0.058 (2)	0.062 (2)	0.051 (2)	0.0063 (18)	0.0032 (16)	−0.0289 (18)
C5	0.0349 (16)	0.0487 (18)	0.0323 (14)	−0.0123 (14)	0.0022 (12)	−0.0133 (13)
C6	0.0298 (15)	0.0532 (19)	0.0489 (18)	−0.0094 (14)	−0.0038 (13)	−0.0191 (15)
C7	0.0344 (16)	0.0476 (18)	0.0387 (16)	−0.0007 (13)	−0.0117 (12)	−0.0179 (14)
C8	0.0398 (17)	0.0489 (18)	0.0277 (14)	−0.0074 (14)	−0.0039 (12)	−0.0125 (13)
C9	0.0293 (14)	0.0353 (15)	0.0289 (13)	−0.0068 (11)	−0.0014 (10)	−0.0129 (11)
C10	0.0299 (14)	0.0421 (16)	0.0272 (13)	−0.0056 (12)	0.0013 (10)	−0.0117 (12)
C11	0.0327 (15)	0.0406 (16)	0.0362 (15)	−0.0027 (12)	0.0003 (11)	−0.0171 (13)
C12	0.0366 (16)	0.0317 (16)	0.0494 (18)	−0.0040 (13)	0.0041 (13)	−0.0100 (13)
C13	0.052 (2)	0.055 (2)	0.0358 (16)	−0.0099 (16)	0.0131 (14)	−0.0090 (15)
C14	0.058 (2)	0.068 (2)	0.0357 (16)	−0.0142 (18)	0.0102 (14)	−0.0261 (16)
C15	0.0450 (18)	0.0476 (18)	0.0385 (16)	−0.0129 (15)	0.0054 (13)	−0.0210 (14)
C16	0.065 (3)	0.047 (2)	0.080 (3)	−0.0220 (19)	0.006 (2)	−0.0163 (19)

### Geometric parameters (Å, °)

**Table:**

Cu1—O1	1.9762 (19)	C4—H4C	0.9600
Cu1—O2^i^	1.9866 (19)	C5—C6	1.372 (4)
Cu1—O3	1.967 (2)	C5—H5	0.9300
Cu1—O4^i^	1.966 (2)	C6—C7	1.372 (4)
Cu1—N1	2.197 (2)	C6—H6	0.9300
Cu1—Cu1^i^	2.6532 (6)	C7—C8	1.370 (4)
O1—C1	1.246 (3)	C7—H7	0.9300
O2—C1	1.259 (3)	C8—C9	1.393 (4)
O2—Cu1^i^	1.9866 (19)	C8—H8	0.9300
O3—C3	1.261 (3)	C10—C11	1.387 (4)
O4—C3	1.250 (4)	C10—C15	1.385 (4)
O4—Cu1^i^	1.966 (2)	C11—C12	1.384 (4)
N1—C9	1.339 (3)	C11—H11	0.9300
N1—C5	1.352 (3)	C12—C13	1.385 (4)
N2—C9	1.370 (3)	C12—C16	1.503 (4)
N2—C10	1.413 (3)	C13—C14	1.377 (5)
N2—H2	0.8600	C13—H13	0.9300
C1—C2	1.498 (4)	C14—C15	1.380 (4)
C2—H2A	0.9600	C14—H14	0.9300
C2—H2B	0.9600	C15—H15	0.9300
C2—H2C	0.9600	C16—H16A	0.9600
C3—C4	1.500 (4)	C16—H16B	0.9600
C4—H4A	0.9600	C16—H16C	0.9600
C4—H4B	0.9600		
			
O4^i^—Cu1—O3	167.64 (8)	H4A—C4—H4C	109.5
O4^i^—Cu1—O1	88.77 (9)	H4B—C4—H4C	109.5
O3—Cu1—O1	90.33 (9)	N1—C5—C6	123.4 (3)
O4^i^—Cu1—O2^i^	89.16 (9)	N1—C5—H5	118.3
O3—Cu1—O2^i^	89.01 (9)	C6—C5—H5	118.3
O1—Cu1—O2^i^	167.27 (8)	C5—C6—C7	118.1 (3)
O4^i^—Cu1—N1	98.40 (8)	C5—C6—H6	121.0
O3—Cu1—N1	93.96 (8)	C7—C6—H6	121.0
O1—Cu1—N1	94.60 (8)	C8—C7—C6	119.8 (3)
O2^i^—Cu1—N1	98.13 (8)	C8—C7—H7	120.1
O4^i^—Cu1—Cu1^i^	84.47 (6)	C6—C7—H7	120.1
O3—Cu1—Cu1^i^	83.19 (6)	C7—C8—C9	119.3 (3)
O1—Cu1—Cu1^i^	83.69 (6)	C7—C8—H8	120.3
O2^i^—Cu1—Cu1^i^	83.61 (6)	C9—C8—H8	120.3
N1—Cu1—Cu1^i^	176.65 (6)	N1—C9—N2	115.0 (2)
C1—O1—Cu1	124.57 (18)	N1—C9—C8	121.4 (2)
C1—O2—Cu1^i^	123.80 (18)	N2—C9—C8	123.6 (2)
C3—O3—Cu1	124.0 (2)	C11—C10—C15	119.4 (3)
C3—O4—Cu1^i^	122.82 (18)	C11—C10—N2	117.7 (2)
C9—N1—C5	118.0 (2)	C15—C10—N2	122.7 (3)
C9—N1—Cu1	127.85 (17)	C10—C11—C12	121.6 (3)
C5—N1—Cu1	114.18 (17)	C10—C11—H11	119.2
C9—N2—C10	127.9 (2)	C12—C11—H11	119.2
C9—N2—H2	116.1	C13—C12—C11	118.2 (3)
C10—N2—H2	116.1	C13—C12—C16	121.2 (3)
O1—C1—O2	124.3 (2)	C11—C12—C16	120.6 (3)
O1—C1—C2	117.9 (2)	C14—C13—C12	120.5 (3)
O2—C1—C2	117.8 (3)	C14—C13—H13	119.8
C1—C2—H2A	109.5	C12—C13—H13	119.8
C1—C2—H2B	109.5	C13—C14—C15	121.2 (3)
H2A—C2—H2B	109.5	C13—C14—H14	119.4
C1—C2—H2C	109.5	C15—C14—H14	119.4
H2A—C2—H2C	109.5	C14—C15—C10	119.1 (3)
H2B—C2—H2C	109.5	C14—C15—H15	120.5
O4—C3—O3	125.3 (3)	C10—C15—H15	120.5
O4—C3—C4	118.0 (3)	C12—C16—H16A	109.5
O3—C3—C4	116.7 (3)	C12—C16—H16B	109.5
C3—C4—H4A	109.5	H16A—C16—H16B	109.5
C3—C4—H4B	109.5	C12—C16—H16C	109.5
H4A—C4—H4B	109.5	H16A—C16—H16C	109.5
C3—C4—H4C	109.5	H16B—C16—H16C	109.5
			
O4^i^—Cu1—O1—C1	−84.3 (2)	C9—N1—C5—C6	−1.4 (4)
O3—Cu1—O1—C1	83.4 (2)	Cu1—N1—C5—C6	178.8 (2)
O2^i^—Cu1—O1—C1	−3.6 (6)	N1—C5—C6—C7	1.3 (5)
N1—Cu1—O1—C1	177.4 (2)	C5—C6—C7—C8	−0.5 (5)
Cu1^i^—Cu1—O1—C1	0.3 (2)	C6—C7—C8—C9	−0.1 (5)
O4^i^—Cu1—O3—C3	0.2 (6)	C5—N1—C9—N2	−177.5 (3)
O1—Cu1—O3—C3	−85.6 (2)	Cu1—N1—C9—N2	2.2 (4)
O2^i^—Cu1—O3—C3	81.7 (2)	C5—N1—C9—C8	0.7 (4)
N1—Cu1—O3—C3	179.8 (2)	Cu1—N1—C9—C8	−179.6 (2)
Cu1^i^—Cu1—O3—C3	−2.0 (2)	C10—N2—C9—N1	175.0 (3)
O4^i^—Cu1—N1—C9	58.8 (2)	C10—N2—C9—C8	−3.2 (5)
O3—Cu1—N1—C9	−121.1 (2)	C7—C8—C9—N1	0.0 (4)
O1—Cu1—N1—C9	148.2 (2)	C7—C8—C9—N2	178.1 (3)
O2^i^—Cu1—N1—C9	−31.6 (2)	C9—N2—C10—C11	−134.6 (3)
O4^i^—Cu1—N1—C5	−121.5 (2)	C9—N2—C10—C15	50.2 (5)
O3—Cu1—N1—C5	58.6 (2)	C15—C10—C11—C12	−0.2 (4)
O1—Cu1—N1—C5	−32.0 (2)	N2—C10—C11—C12	−175.6 (3)
O2^i^—Cu1—N1—C5	148.2 (2)	C10—C11—C12—C13	−2.0 (4)
Cu1—O1—C1—O2	−2.2 (4)	C10—C11—C12—C16	175.8 (3)
Cu1—O1—C1—C2	178.1 (2)	C11—C12—C13—C14	2.4 (5)
Cu1^i^—O2—C1—O1	3.4 (4)	C16—C12—C13—C14	−175.4 (3)
Cu1^i^—O2—C1—C2	−176.9 (2)	C12—C13—C14—C15	−0.6 (5)
Cu1^i^—O4—C3—O3	−5.7 (4)	C13—C14—C15—C10	−1.6 (5)
Cu1^i^—O4—C3—C4	174.4 (2)	C11—C10—C15—C14	2.0 (4)
Cu1—O3—C3—O4	5.2 (4)	N2—C10—C15—C14	177.2 (3)
Cu1—O3—C3—C4	−175.0 (2)		

Symmetry codes: (i) −*x*+1, −*y*+1, −*z*+1.

### Hydrogen-bond geometry (Å, °)

**Table:**

*D*—H···*A*	*D*—H	H···*A*	*D*···*A*	*D*—H···*A*
N2—H2···O2^i^	0.86	2.17	2.913 (3)	145

Symmetry codes: (i) −*x*+1, −*y*+1, −*z*+1.

## References

[bb1] Barbour, L. J. (2001). *J. Supramol. Chem.* **1**, 189–191.

[bb2] Barquín, M., González Garmendia, M. J., Pacheco, S., Pinilla, E., Quintela, S., Seco, J. M. & Torres, M. R. (2004). *Inorg. Chim. Acta*, **357**, 3230–3236.

[bb3] Bruker (2008). *APEX2* and *SAINT*. Bruker AXS Inc., Madison, Wisconsin, USA.

[bb4] Seco, J. M., González Garmendia, M. J., Pinilla, E. & Torres, M. R. (2004). *Polyhedron*, **21**, 457–464.

[bb5] Sheldrick, G. M. (1996). *SADABS*. University of Göttingen, Germany.

[bb6] Sheldrick, G. M. (2008). *Acta Cryst.* A**64**, 112–122. 10.1107/S0108767307043930 18156677

[bb7] Sieroń, L. (2004). *Acta Cryst.* E**60**, m577–m578.

[bb8] Westrip, S. P. (2009). *publCIF*. In preparation.

